# Supplementary material for: Modeling the influence of temperature and water potential on seed germination of Allium tenuissimum L
Source: PeerJ. 2020 Apr 7;8:e8866. doi: 10.7717/peerj.8866 (PMC7147430; doi:10.7717/peerj.8866)
Supplement: Table S1 — T, temperature; Ψ, water potential; T ×Ψ, the interaction of temperature and water potential; df, degree of freedom [file peerj-08-8866-s002.docx]

**Table S1** ANOVA analysis for the final germination percentage of *A. tenuissimum* seeds incubated at various temperatures and water potential conditions.

| Source | df | Mean Square | *F* value | *P* value |
| --- | --- | --- | --- | --- |
| *T* | 4 | 10000.3 | 587.9 | <0.0001 |
| *Ψ* | 3 | 10938.3 | 643.0 | <0.0001 |
| *T*×*Ψ* | 12 | 411.6 | 24.2 | <0.0001 |

*T*, temperature; *Ψ*, water potential; *T*×*Ψ*, the interaction of temperature and water potential; df, degree of freedom
